# Individual variability in habitat selection by aquatic insects is driven by taxonomy rather than specialisation

**DOI:** 10.1038/s41598-022-25363-3

**Published:** 2022-12-01

**Authors:** Hana Šigutová, Martin Šigut, Aleš Dolný, Filip Harabiš

**Affiliations:** 1 Department of Biology and Ecology, Faculty of Science, University of Ostrava, Ostrava, Czech Republic; 2 Department of Zoology, Faculty of Science, Palacký University, Olomouc, Czech Republic; 3 Department of Ecology, Faculty of Environmental Sciences, Czech University of Life Sciences, Prague, Czech Republic

**Keywords:** Ecology, Zoology

## Abstract

Habitat selection, the choice of a habitat based on its perceived quality, is a key mechanism structuring freshwater communities. To date, individual variability in habitat selection has been neglected, and specialisation has never been considered in this type of studies. We examined the individual differences in the habitat selection of backswimmers (Notonectidae) and diving beetles (Dytiscidae). From each family, we selected one habitat generalist able to coexist with fish (*Notonecta glauca*, *Dytiscus marginalis*), and one species specialised to fishless habitats (*Notonecta obliqua*, *Acilius sulcatus*). We performed a mesocosm experiment quantifying the consistency in individuals’ decisions in response to fish and vegetation structure, in relation to sex and specialisation. Neither the overall pattern of preferences nor consistency in individuals’ decisions differed between specialists and generalists or between the sexes, but both were consistent within families. At the population level, backswimmers preferred fishless pools with submersed and floating macrophytes, while diving beetles showed no clear preferences. Individual decisions of backswimmers were consistent and likely driven by conspecific/heterospecific attraction. In diving beetles, individual decisions were primarily density-dependent. Our results reinforce the significance of habitat selectivity for aquatic community assembly, while suggesting a range of mechanisms driving variability in individual behaviour.

## Introduction

Among the various types of animal behaviour, habitat selection, the choice of a patch based on its perceived quality^1^, is increasingly being acknowledged as a key mechanism responsible for structuring aquatic populations, communities, and metacommunity assemblages^2–4^. Habitat selection is closely linked to niche differentiation and can even transcend the impact of post-colonisation processes, such as non-random site-specific competition, resource-related mortality, and predation^2,3^. As this behaviour brings individuals to the resources essential for survival and reproduction, understanding the factors shaping individuals’ decisions is an ongoing challenge^5,6^.

Habitat selection is a complex process, and is based on specific visual, tactile, and chemical cues, or a combination thereof. In aquatic environments, one of the most important factors determining the suitability of habitat for colonisation by invertebrate communities is the presence of predatory fish^7,8^. Because vulnerability and behavioural responses to specific predators vary among prey species^9–11^, the intensity of their behavioural responses should correlate with their vulnerability to predation^12^. Other important factors affecting habitat selection include vegetation structure^13,14^ and competition for space and resources^15–17^.

Throughout their lives, animals are repeatedly required to make decisions regarding resource selection (e.g., prey, refuges, oviposition sites) in order to maximise their fitness^18^. At instances where individual variability in habitat selection covaries with fitness, such variability may represent an alternative strategy for adaptive evolution^5,16,19^. Therefore, understanding the causes and consequences of individual variability in habitat selection within a population is crucial to answering key ecological and evolutionary questions, such as the extent to which individuals develop specialised niches that are narrower than those of the population, which may in turn lead to rapid diversification^20,21^. However, the potential for evolution to act on individual variation in habitat selection behaviour remains unexplored, and investigating the consistency in animal decisions is the first step towards answering this question^5^.

Although there is considerable individual variation in various types of animal preferences, both spatially and temporally^22,23^, it has been largely ignored in habitat selection studies. However, experiments with vertebrates have revealed that individual variation may be obviated by group patterns, and thus may not be apparent at the population level^5,24,25^. This may arise, among other factors, due to contrasting responses linked to individual characteristics, such as sex, age, or body condition^25^, or density-dependent habitat selection^26^ when animals may favour an ideal free distribution to minimise competition and maximise their fitness^27^.

However, most studies have quantified habitat selection at the level of a whole population or assemblage, as applied to vertebrates^28–30^ and invertebrates^2,12,29^. To the best of our knowledge, all studies on individual variability in habitat selection behaviour have focused on vertebrates, mainly fish, birds, and large mammals^5,19,24,25,31–35^. Insects have been ignored in this context, even though they are good model organisms for ecological and evolutionary research^36,37^. Semi-aquatic insects in particular, with their short and complex life cycles, aquatic larval stages, and winged mobile imagoes which colonise new habitats, are likely to exhibit habitat selectivity^38^. They exhibit behavioural plasticity and associative learning^39–41^, making them an ideal group for studying individual variability in habitat selection. Furthermore, insects represent an important comparison to behavioural studies of vertebrates, as both groups have independently evolved many complex behaviours^42^. Therefore, identification of differences and similarities in individual variation in habitat selection of insects and vertebrates can help understand the generality of the patterns found in vertebrates. Last but not least, unlike vertebrates, studies involving insects are usually free of ethical issues associated with their manipulation.

In our previous study^11^, we focused on the population-level differences in habitat selection between specialists and generalists of semiaquatic and aquatic insects and amphibians. In the present study, we focused on two groups of aquatic insects from our dataset: backswimmers (Notonectidae), and diving beetles (Dytiscidae). We investigated individual differences in their habitat selection behaviour in relation to sex and the level of specialisation. From each family, one species was a habitat generalist that was able to coexist with fish (*Notonecta glauca*, *Dytiscus marginalis*), and one species was specialised to live in fishless habitats (*Notonecta obliqua, Acilius sulcatus*). In a mesocosm experiment, we tracked responses of marked individuals to the non-consumptive presence of fish and vegetation structures mimicking different successional stages of aquatic habitats. We aimed to determine (1) sex-related differences in the overall habitat preferences, (2) frequency of changes in habitat preferences of individuals over time, and (3) consistency in individuals’ decisions. We hypothesised that overall habitat preferences and consistency in individuals’ decisions would differ between habitat specialists and generalists. Specialists, who have specific habitat requirements and are vulnerable to fish predation owing to the absence of defensive or compensatory mechanisms^43–45^, would show clear preferences and, therefore, a significant intra- and inter-individual consistency would be observed in individuals’ habitat selection decisions. Generalists would show neither clear preferences for a specific habitat type nor decision consistency as they may coexist with fish^45,46^, and therefore may inhabit a wide range of habitats. By exploring the consistency in individuals’ decisions and habitat preferences at the population level, we were able to disentangle the role of habitat characteristics in habitat selection (fish and vegetation structure) from information from other sources, such as the presence of conspecifics and heterospecifics.

## Methods

### Study species

In our division of species to generalists and specialists, we worked on the presumption that specialists to fishless habitats should be more susceptible to fish predation (due to intrinsic traits—e.g., body size, level of activity, presence of defensive mechanisms) and should prefer fishless habitats (e.g., acidic bogs or habitats in early successional stages).

The generalist backswimmer was represented by *Notonecta glauca* Linnaeus, 1758 (Hemiptera: Notonectidae) (13–16 mm) which inhabits a wide range of habitats. Due to its smaller body size and lighter colour it may coexist with fish^44^, and is frequently found in habitats with fish^46^. The more specialised *Notonecta obliqua* Thunberg, 1787 (14–17 mm) prefers acidic bogs and fens^47,48^. Due to its larger body size and dark colouration, it is highly susceptible to fish predation^44^, and thus likely shows a preference for fishless habitats.

The large diving beetle *Dytiscus marginalis* Linnaeus, 1758 (Coleoptera: Dytiscidae), inhabits various aquatic habitats, including those inhabited by fish. Its coexistence with fish is enabled by its large body (27–35 mm), short larval stage, defensive secretions, and hard cuticle^45^. The smaller *Acilius sulcatus* Linnaeus, 1758 (15–18 mm) is mostly found in larger water bodies with rich submersed vegetation^49^, but may also colonise temporary habitats or those in early successional stages to avoid fish^50,51^. Owing to their smaller size, both larvae and adults are susceptible to predation^52^, and adults respond to fish chemical stimuli^50^.

### Experimental design

The present study was a part of a mesocosm experiment aimed at understanding habitat selection of (semi)aquatic insects and amphibians^11^ whose data have been re-analysed to account for the individual codes of the study animals. The experiment consisted of 24 plastic tanks (275 L, 1,1 m diameter, 35 cm depth) arranged in four blocks, each of six pools with different combination of predator presence and vegetation type, randomly assigned within a block (for details, see^11^). Individual blocks consisted of four outdoor net cages, each of 12 × 6 × 3 m (steel construction covered with polyamide netting with 2 × 2 mm mesh size), located in the botanical garden of the University of Ostrava, Czech Republic (49.8274 N, 18.3259 E). The tanks were filled with well water and were surrounded by grass.

All tanks received plastic predator cages (40 cm diameter × 40 cm height) covered with a polyethylene screen with 5 × 5 mm mesh size. This allowed larger prey to pass through the cage while providing visual and chemical cues to experimental organisms, but preventing fish from consuming them. Fish were represented by thee 15–20 cm long individuals tof the crucian carp *Carassius auratus* (Cyprinidae) which is an invasive, omnivorous predator of aquatic insects, typically found in European lentic habitats^53^. Backswimmers and diving beetles were sampled according to a fully randomised 2 × 3 factorial design (fish or fishless pools × no macrophytes; only submerged and floating macrophytes; submersed, floating, and littoral macrophytes). Submersed and floating macrophytes (*Potamogeton natans, Nuphar lutea, Nymphaea alba, Trapa natans*, and *Elodea canadensis*) were distributed evenly throughout the particular pools. Littoral (emergent) macrophytes (*Juncus* spp., *Carex* spp. *Iris pseudacorus*, and *Eleocharis palustris*) were distributed along the edges of the particular pools (see Supplementary Fig. S1 online). Composition, levels, and arrangement of the vegetation was similar in the fish and fishless pools. For details concerning the provision of food to fish and experimental organisms see^11^.

### Data sampling

Diving beetles were experimentally sampled from the mesocosms from 27 May to 30 June 2019. Diving beetles exhibit strong sexual dimorphism; we used grooves on the female elytra and suction cups on male tarsomers to distinguish males from females^54^. At the beginning of the experiment, 52 *D. marginalis* individuals were released into two blocks (26 per block; 16 males, 10 females), and 106 *A. sulcatus* in the other two blocks (53 per block; 30 males, 23 females) to prevent predation of *A. sulcatus* by *D. marginalis*^55^. Ensuring that each species experienced a block was more important than having 11 consecutive sampling events in the same blocks because there could have been a significant effect of a block on insect behaviour. Before the initial release, each individual was marked on the elytra using a permanent marker and a unique code (see Supplementary Fig. S1 online). Subsequently, all beetles were divided randomly into three equally numerous groups. Each group was released onto one of three trays (20 × 20 cm) placed between each pair of pools within a block. The trays contained a small amount of water to promote the dispersal to our experimental pools. Individual habitat selection decisions were observed approximately every three days, resulting in 11 sampling events. During each event, all macrophytes were removed and pools were carefully checked for beetles using hand nets (0.5 cm and 1 mm mesh). Beetles were counted, and their individual codes were noted. Beetles were then transferred to a single container, and after examining all pools within a block, they were released following the same procedure as during initial release to allow for de novo selection. After the fifth sampling, all *A. sulcatus* individuals were relocated to the two blocks originally inhabited by *D. marginalis* and vice versa*,* to ensure a balanced experimental design (i.e., each species was sampled in all four blocks).

Backswimmers were sampled from 8 August to 9 September 2019. Backswimmers exhibit only minor sexual dimorphism in size and colour^56,57^, therefore, the shape of the ventral abdominal segments was used to distinguish between males and females^58^. The blocks were stocked with 80 N*. glauca* individuals (20 per block; 8 males, 12 females) and 88 N*. obliqua* individuals (22 per block; 11 males, 11 females). Intrageneric predation is unlikely among similar-sized backswimmers^59^, and both species commonly coexist in nature^46^. Therefore, they were simultaneously kept in all blocks. Sampling protocol followed that of diving beetles, except for the species switch, which was unnecessary in this case.

### Data analysis

To analyse overall habitat preferences, we used the generalised estimating equations (GEE) approach for fitting marginal generalised linear models to clustered data using the geeglm function, as our data represented repeated observations of preferences of the same individuals. The geeglm function has a similar syntax as glm and returns a similar object but allows the specification of the correlation structure of datasets. The geeglm function fits generalised estimating equations using the 'geese.fit' function of the 'geepack' package^60^. In our models with a binomial distribution of the errors (link = logit) and exchangeable correlation structure. Because the numbers of individuals entering the experiments were different across species and sexes, our response variable was the proportion of individuals that preferred individual pools. *Habitat type* (six combinations; fish or fishless pools × no macrophytes; only submerged and floating macrophytes; submersed, floating, and littoral macrophytes) was the independent variable. To identify sex differences in habitat preferences, we included the interaction *Sex : Habitat type* as an independent variable in each model. The identification of the pool (id) was used for the specification of individual clusters. An analysis of variance that compares models through Wald tests was used to get the most parsimonious model. We considered a non-preferred habitat as the type that was occupied by less than a total of 15% of individuals. Package 'lsmeans'^61^ with Tukey contrasts was used for the pair-wise comparison between individual habitat types.

For the remaining analyses, only individuals that were captured in at least half of all the sampling events were used. For the general comparison of habitat change frequency (a proportion of individuals changing habitat type between two subsequent sampling events), mixed models (GLMMs) with a binomial distribution of errors were performed using the ‘lme4’ package^62^. In these models, the dependent variable was always the ratio of individuals who changed their habitat to those whose preferences remained unchanged, whereas *Sex* and *Sampling event* were used as fixed effects. Block was used as a random effect. We also used generalized linear models (GLM) with a binomial distribution of errors to analyse the proportion of individuals changing habitat type after their initial preference (only in relation to fish presence, i.e., switch from fishless to fish-containing habitats and vice versa). In these models, the dependent variable was the ratio of individuals that changed their habitat to those whose preferences remained unchanged, and Initial *Habitat* choice (fish-containing, fishless) and *Sex : Habitat* were used as fixed effects.

In repeatability analysis we aimed to measure the random intra- and inter-individual variability. More precisely, we focused on an extent to which an individual stuck to its favourite habitat type (i.e., habitat type which an individual selected most frequently; intra-individual consistency), and examined whether there are differences in selection consistency among individuals. We used the R package 'rptR' for binomial-distributed data to test intra-individual variability in decision making in repeated measures (repeatability, R), i.e., what is the proportion of random individual variability versus repeatability of fixed effects (*Sampling event* and *Sex*). Uncertainty in estimators was quantified by parametric bootstrapping, and significance testing was implemented by likelihood ratio tests and through permutation of residuals. The package 'rptR' allows to control for fixed effects, and thus to estimate the adjusted repeatability (that removes fixed effect variance from the estimate) and enhanced agreement repeatability^63^. All analyses were performed in R version 4.0.2^64^. The statistical significance level was set as 0.05.

### Ethics declaration

The study on animals approved the Institutional Animal Care and Use Committee of the Institute of Animal Physiology and Genetics CAS, v.v.i., Libechov, Czech Republic, in agreement with the joint research workplace and the contractual cooperation partnerships. Animals were handled by Lukas Choleva (see acknowledgements) awarded the Certificate of competency according to §17 of the Czech Republic Act No. 246/1992 coll. on the Protection of Animals against Cruelty (Registration number CZ 02361), provided by the Central Commission for Animal Welfare (the Ministry of Agriculture of the Czech Republic). The study was conducted in compliance with ARRIVE guidelines. Fish handling was carried out according to the European Union Directive (86/609EEC) for the protection of animals used for experimental and other scientific purposes, and the “Guidelines for the treatment of animals in behavioural research and teaching”. No mortality or stress was observed. Fish were sourced from a local fishery; at the end of the study, they were released into local ponds. No specific permissions were required to collect and handle insects. The taxa in use are common species, they are not endangered or protected by law in the Czech Republic, or species at the risk of extinction according to the IUCN Policy Statement on Research Involving Species at Risk of Extinction.

## Results

### Overall habitat preferences

**Figure 1:**
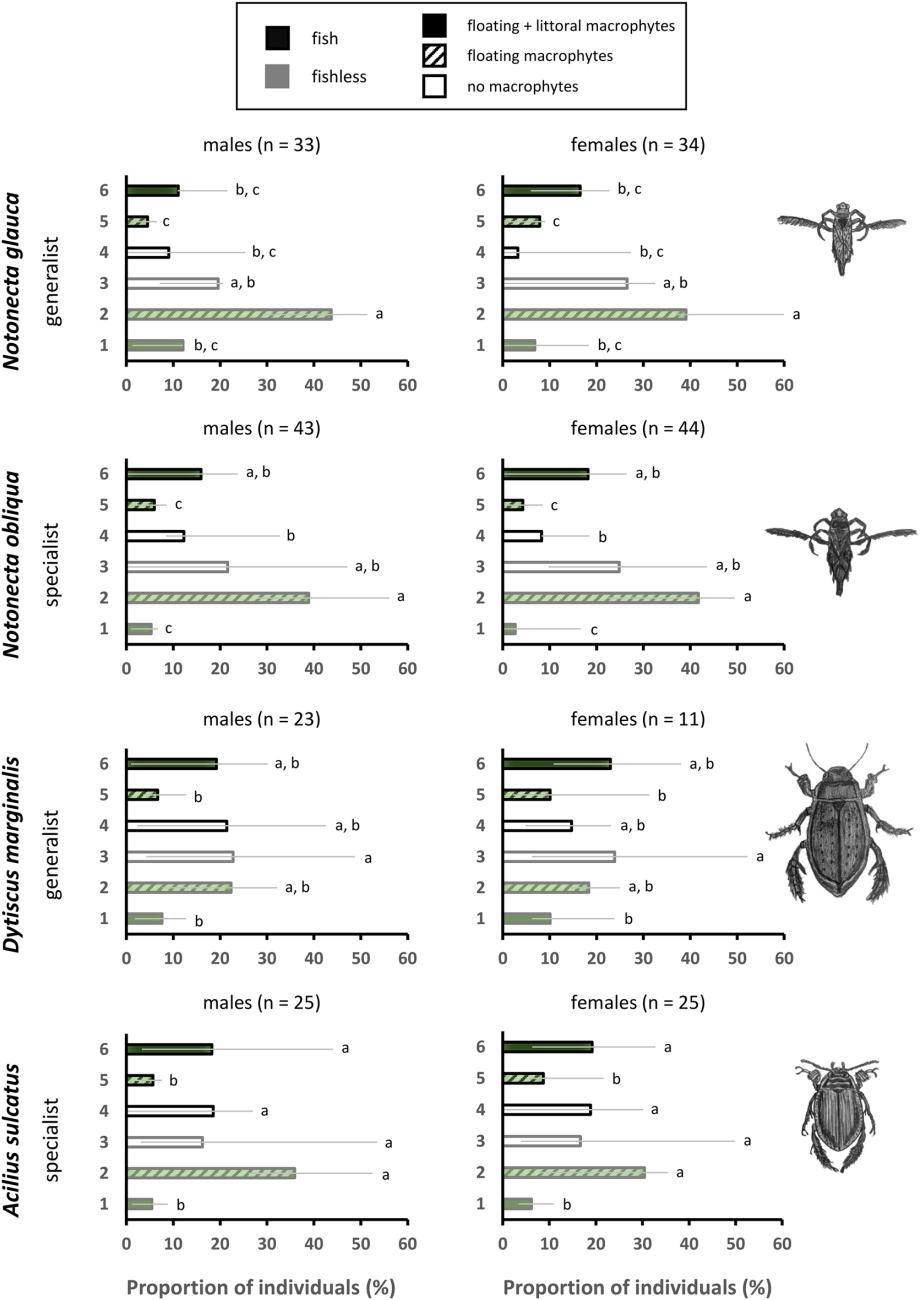
Overall habitat preferences of the study taxa. Proportion of occurrences in six habitat types (fish and fishless pools without macrophytes, with submersed and floating macrophytes, or with submersed, floating plus littoral macrophytes) in backswimmers (*Notonecta glauca*, *N. obliqua*) and diving beetles (*Dytiscus marginalis*, *Acilius sulcatus*). *Notonecta glauca* and *D. marginalis* represent habitat generalists, while *N. obliqua* and *A. sulcatus* are species specialised for life in fishless habitats, as they are vulnerable to fish predation. Error bars show the variance in preferences of individuals for each habitat type among individual blocks; n is the number of individuals entering the analysis.

Both backswimmer species showed significant habitat preferences (*Notonecta glauca*: χ^2^_5_ = 117.0, *P* < 0.001; *N. obliqua*: χ^2^_5_ = 87.2, *P* < 0.001), and were most frequently found in fishless habitats with submersed and floating macrophytes (Fig. 1). In diving beetles, it was not possible to distinguish the most preferred habitat type; nevertheless, we were able to distinguish the differences between preferred and non-preferred habitat types (*D. marginalis*: χ^2^_5_ = 14.94, *P* = 0.011; *A. sulcatus*: χ^2^_5_ = 64.7, *P* < 0.001). Similar to backswimmers, both beetle species avoided fish-containing pools with submersed and floating macrophytes as well as fishless pools with submersed, floating plus littoral macrophytes; however, they did not differentiate between the other habitat types (Fig. 1). In all study species, there were no differences between the preferences of males and females (*Notonecta glauca*: χ^2^_6_ = 4.00, *P* = 0.068; *N. obliqua*: χ^2^_6_ = 1.70, *P* = 0.95; *D. marginalis*: χ^2^_6_ = 4.64, *P* = 0.590; *A. sulcatus*: χ^2^_6_ = 2.00, *P* = 0.920, Fig. 1).

### Habitat change frequency

**Figure 2:**
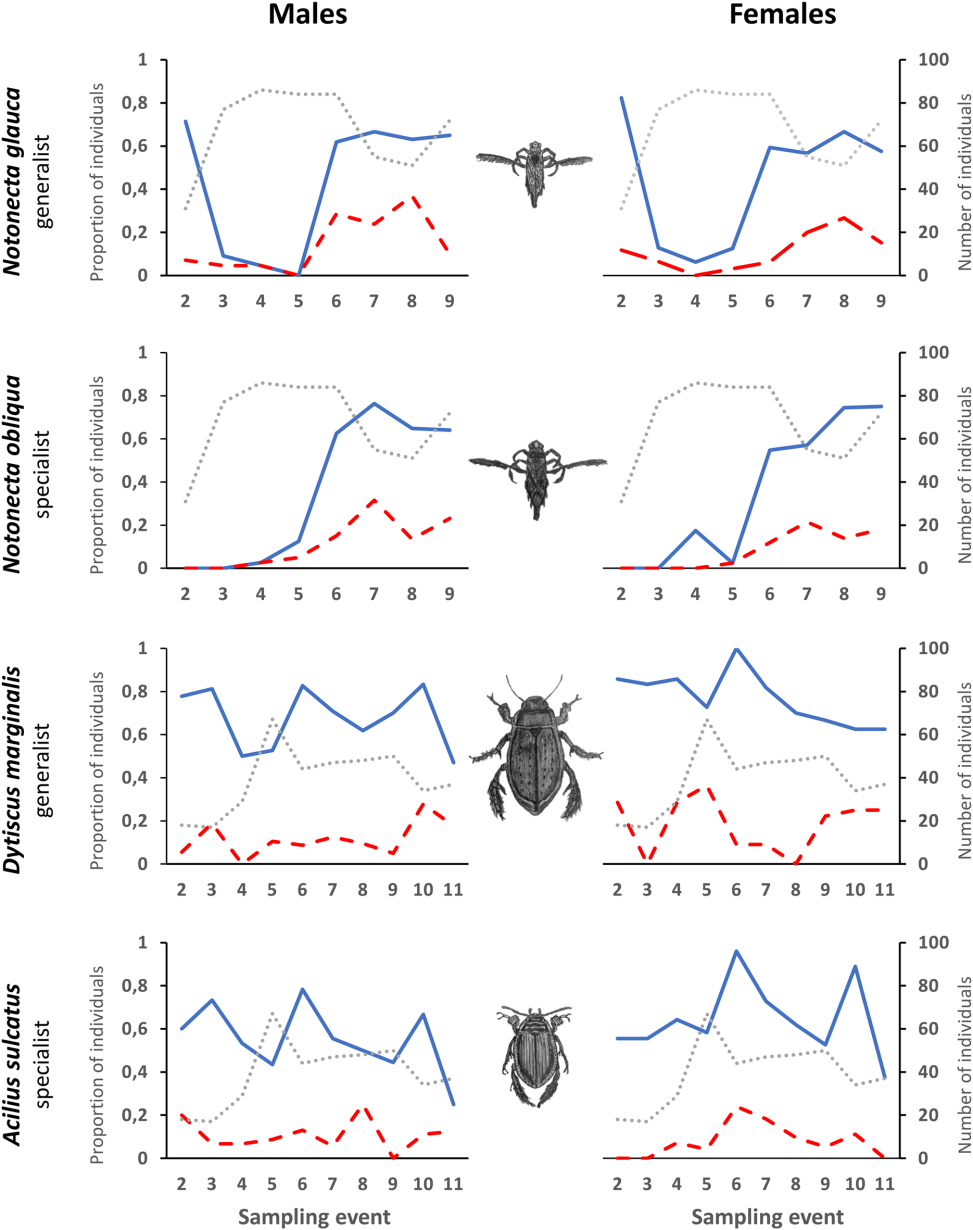
Habitat change frequency over time. Cumulative proportion of individuals changing habitat type between sampling events (blue), and the proportion of individuals opting for non-preferred habitat types (red dashed line; habitat types with occurrence of individuals in less than 15% of all cases) in backswimmers (*Notonecta glauca*, *N. obliqua*), and diving beetles (*Dytiscus marginalis*, *Acilius sulcatus*). Additional lines were included to visualize the total densities of individuals (both sexes) occurring in the most preferred habitat type = dotted line (PH; backswimmers: fishless habitats with submersed and floating macrophytes; diving beetles: the sum of individuals found in all preferred habitats).

Marking individual animals allowed us to determine the changes in their preferences over time. Initially, individuals of both backswimmer species were found, with a few exceptions, in the same habitat type. However, between the fourth and fifth sampling events, their preferences abruptly changed, with the frequency of changes between sampling events increasing to include almost 70% of all individuals (Fig. 2). As a result, there was a significant decrease in the number of individuals in the most preferred habitat type (*N. glauca*: χ^2^ = 49.70, *P* < 0.001; *N. obliqua*: χ^2^ = 112.17, < 0.001), and an increased incidence in previously non-preferred habitats (Fig. 2). This behavioural change was consistent between the sexes (*N. glauca*: χ^2^ = 0.78, *P* = 0.377; *N. obliqua*: χ^2^ = 2.145, *P* = 0.143; see Supplementary Fig. S2 online).

**Figure 3:**
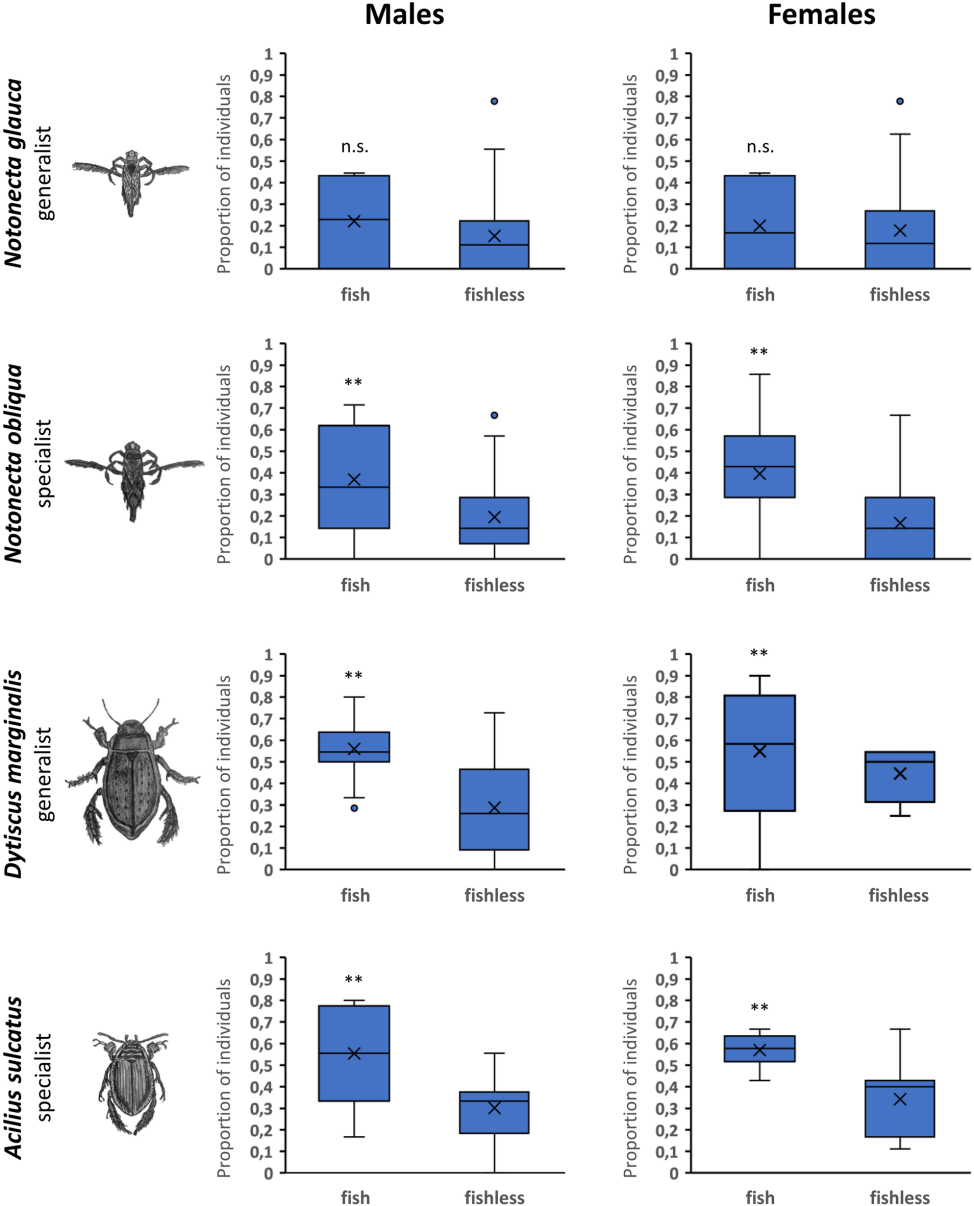
Habitat change frequency in relation to the initial choice. The proportion of individuals who switched the habitat type (from fish-containing to fishless and vice versa) after the first sampling event.

In diving beetles, the frequency of changes was high, but remained more or less constant during the experiment, i.e., changes occurred with the same frequency between individual sampling events (*D. marginalis*: χ^2^ = 1.85, *P* = 0.174; *A. sulcatus*: χ^2^ = 0.026, *P* = 0.871; Fig. 2). In both species, sex-related differences were not significant (*D. marginalis*: χ^2^ = 3.02, *P* = 0.082; *A. sulcatus*: χ^2^ = 0.69, *P* = 0.406; see supplementary Fig. S2 online). The comparison of decision-making mechanisms in relation to the individual’s initial choice regarding the fish presence indicated that in *N. glauca*, only 18% of individuals chose a fish-containing habitat during the first sampling event. However, the frequency of changes of habitat type (switch from fish-containing to fishless habitats and vice versa) throughout the experiment was similar for all individuals, regardless of their initial choice (fish-containing vs fishless habitats) (Dev = 0.14, *P* = 0.510; Fig. 3). In the remaining species, the tendency to select fish-containing habitats as the first option was higher: 36% (*N. obliqua*), 19% (*A. sulcatus*) and even 59% of individuals (*D. marginalis*) were found in fish-containing habitats in the first sampling event. Nevertheless, the tendency of these individuals to switch to the fishless option was significantly higher than was the tendency of individuals with fishless first choice to switch to fish-containing habitats (*N. obliqua*: Dev = 5.31, *P* < 0.001; *D. marginalis*: Dev = 1.74, *P* = 0.007; *A. sulcatus*: Dev = 2.25, *P* < 0.001; Fig. 3). There were no differences in these decision-making mechanisms between the sexes (*N. glauca*: Dev = 0.39, P = 0.82; *N. obliqua*: Dev = 0.08, P = 0.89; *D. marginalis*: Dev = 0.02, P = 0.91; *A. sulcatus*: Dev = 0.09, P = 0.69).

### Individual variability in habitat selection

In both species of backswimmers, most individuals opted for three habitat types, while both beetle species most frequently opted for four habitat types (five in *D. marginalis* females; Supplementary Fig. S3 online). Based on the repeatability analysis (intra-individual variability), only backswimmers stuck to certain habitat types (i.e., individuals exhibited fidelity to their preferred habitats). However, their repeatability was affected by sampling event; the order within repeated measures explained 21.9% of variability in the model for *N. obliqua*, and 16.6% of variability for *N. glauca,* implying that the preferences for a certain habitat type were significantly related to the sampling event (medium size of the effect). In effect, individuals’ decisions were very consistent until they suddenly changed throughout the experiment. Regarding inter-individual variability, estimated repeatability in decision making was significant only in *N. glauca* (P = 0.002 [LRT], 7.3% of explained variability, indicating small size effect of intra-individual variability, and only negligible intra-individual variability in *N. obliqua*: P = 0.423 [LRT]; 0.6% of variability). Contrarily, in diving beetles, when evaluating repeatability in decision making of fixed effect, the order within repeated measures explained only 2.3% of variability for *A. sulcatus*, and 0.3% for *D. marginalis*, suggesting that their behavioral patterns remained random during the experiment. Similar to fixed effect measurements, the intra-individual variability in the repeatability in decision making was random in both species; in other words, estimated intra-individual repeatability was negligible. Regarding inter-individual variability, models of estimated repeatability in decision making for both species explained a negligible amount of variability. The habitat selection history of individuals of all study taxa is shown in Supplementary Fig. S4 online.

## Discussion

Contrary to our hypothesis, neither the overall pattern of habitat preferences nor consistency in individuals’ decisions differed between specialists and generalists, but both were consistent within backswimmers and diving beetles. At the population level, backswimmers preferred fishless habitats with floating macrophytes, whereas beetles showed no clear preferences (see^11^ for detailed discussion on differences in overall habitat preferences).

The conditions of fishless pools with submersed and floating macrophytes comply with the habitat requirements of all study taxa, as these pools were always among the preferred habitat types. In diving beetles, habitat selection was less distinctive in terms of fish presence or vegetation structure. In the large generalist *D. marginalis*, the lack of selectivity between fish and fishless habitats was unsurprising considering its effective defence via secretions produced by the prothoracic and pygidial glands, which are known to have narcotic and toxic effects on fish^45^. The more specialised *A. sulcatus* is vulnerable to fish predation^52^; however, adults decrease their activity only in response to starved perch, and when both visual and chemical predator cues are present in the environment^50^. The array of responses of various beetle species may vary according to fish species^7,65^; moreover, in the present study, the effect of fish was non-consumptive, but in some taxa, signals from devoured conspecifics are necessary to trigger behavioural avoidance of fish-containing habitats^66,67^*.*

Interestingly, fishless pools with submersed, floating, and littoral macrophytes were among the least popular habitat types in all study taxa, whereas their fish-containing counterparts were preferred. This preference might have arisen from the fact that pools with fish, despite having lower prey abundance, may also entail a lower abundance of competitors, and might thus be selected. Habitat selection is a dynamic process; responses to habitats containing predators (selection and avoidance) are modified through taxa-dependent feedbacks^29^ which were not accounted for in our study. The colonisation rate in fish habitats may be further reinforced by the predator dilution effect^68^, whereby pools with conspecifics may attract further individuals to reduce the overall predation risk.

The overall differences in selectivity between backswimmers and diving beetles may further arise from the differences in mobility traits of both groups. Both species of diving beetles are fast swimmers; due to their streamlined bodies, short tibiae and long tarsi, they move actively through open waters, whereas the swimming ability of backswimmers is poorer^69,70^. On the other hand, as the sit-and-wait predators, backswimmers have a remarkable ability of rapid acceleration which enables them to escape from the fish attack, but only when the shelter is available as this strike is possible only over short distances^69^. Contrarily, the acceleration rate of diving beetles is low^12^. These shared characteristics within the groups may have contributed to the shared habitat preferences within backswimmers and diving beetles, and the lack of distinction between specialists and generalists. Other possible mechanisms driving the selection of the study taxa become apparent only after the examination of individuals’ behaviour.

Analysis of changes in individuals’ preferences over time revealed different habitat selection strategies in backswimmers and diving beetles; however, no differences were found between specialists and generalists. In backswimmers, both sexes showed similar patterns; their tendency to change habitat between two subsequent sampling events remained quite low in the first half of the experiment, despite the accumulation of individuals in the preferred habitat (fishless pool with submersed and floating macrophytes). After the fourth sampling event, however, the tendency to choose different habitats sharply increased, when almost 70% and 80% of individuals (*N. glauca* and *N. obliqua*, respectively) abruptly changed their choice, along with the tendency to opt for previously overlooked habitat types. This behavioural change might result from the depletion of food sources in the preferred type^26^. As the food supply was not supplemented, the frequency of changes remained constant until the end of the experiment.

In contrast, in diving beetles, the number of individuals that changed habitat type between two subsequent sampling events remained high throughout the experiment, and was similar in both species. In males, habitat change frequency oscillated between 50 and 80%, whereas in females, it ranged from 60% to almost 100%; however, the differences in habitat change frequency of males and females were not significant. Unlike in backswimmers, in diving beetles, an increase in density in the preferred habitats was followed by an increase in the tendency to change habitats. This pattern is indicative of the higher involvement of density-dependent habitat selection in diving beetles. The theory of density-dependent habitat selection assumes that individuals should be distributed relative to the profitability of the habitat; with an increasing density in high-quality habitats, individuals are forced to occupy lower-quality habitats^71^. In backswimmers, the observed pattern is more indicative of a “spillover” pattern of the source-sink theory^72^, as individuals presumably first occupied the habitat of the highest quality, prior to switching to lower-quality habitats after forage depletion^26^.

The behaviour of backswimmers detected in this study may be further explained by their temporal life history and coexistence patterns. Both species overwinter as adults and reproduce in spring^46,73^ following dispersal from their overwintering sites^74^. If adults of aquatic insects have a limited breeding period, they may use the presence of conspecifics or heterospecifics as an indirect cue to select habitat^75,76^. Apart from the indirect indication of habitat quality, the presence of other individuals within a habitat might offer protection against predators by means of communal defence and alarming behaviour, and thus increase the chances of encountering mates^77^. This strategy may be common in nature, although it may not be used by all individuals in the population^75^. Both species of backswimmers in this study commonly coexist within their habitat, as they have slightly different feeding strategies and show different spatial niches^78^. Our results suggest that both species may have displayed conspecific/heterospecific attraction^75,77,79^, at least at the beginning of the experiment when food resources in high-quality habitats were abundant.

The results of the analysis of habitat change frequency complied with the analysis of consistency in individuals’ decisions. Although at the population level both backswimmer species preferred fishless pools with submersed and floating macrophytes, some individuals opted for this habitat type more frequently than others, and only a few individuals were faithful to only one habitat type during the experiment; most individuals ranged among three different habitat types (Supplementary Fig. S3 online). Based on the repeatability analysis, individuals of both species were consistent in their habitat selection behaviour. However, when we tracked the individuals’ selection history, it was not possible to draw general conclusions about cues used for habitat selection, as their decisions were quite inconsistent in terms of fish presence or preferred vegetation structure (see Supplementary Fig. S4 online). Moreover, the consistency in the backswimmers’ decisions was significantly affected by sampling event, altogether pointing out the importance of the aforementioned life history and coexistence patterns for habitat selection. Except for *Notonecta glauca*, the decisions of individuals of all study species were affected by their initial habitat choice. Individuals that selected fishless pools as their first habitat had a lower tendency to switch to the fish-containing pools. This finding reaffirms the importance of the associative learning in insect behaviour^7,39,40^.

From all study species, only *N. glauca* exhibited significant inter-individual variability in the repeatability. In other words, certain individuals were very consistent and their preferences did not change over time, whereas in *N. obliqua*, almost all individuals changed their preferred habitat type at some point. The presence of inter-individual variability in the generalist *N. glauca* was not surprising; generalist backswimmer species may exhibit behavioural plasticity in response to changes in ecological factors, such as the presence of predator semiochemicals^80^, whereas the constraints of specialists (morphological, behavioural, or life-history) may substantially reduce their survival in various habitat types. Consequently, generalist species may thrive in various environments which may lead to inter-individual variability in their habitat selection. This may arise from a vast array of mechanisms, such as functional variation in intrinsic traits (e.g., morphology or experience), and may lead to individual specialization. Inter-individual variability is an important target for natural selection as individuals within the population may be subject to various selective pressures^20^; individuals able to use a different niche efficiently have higher fitness as they experience reduced inter- and intraspecific competition^81^. Experiments to test for biomechanical, physiological or cognitive trade-offs that limit individual niche width are necessary to identify the mechanisms that trigger variability in individual behaviour^20^.

Compared to backswimmers, diving beetles were not only less distinctive in their general preferences, but also opted for more habitat types during the experiment, as most individuals ranged among four habitat types (five in *D. marginalis* females); some individuals were found in all six habitat types, while no individual was faithful to only one habitat. Although the number of habitat types selected by males was similar to that of females, they often returned to their preferred habitats (see Supplementary Fig. S4 online). Altogether with the analysis of habitat change frequency, these results suggest that behaviour of males tended to be more consistent, whereas female behaviour was rather random. However, in none of the studied species, the differences in the behaviour of males and females were significant. This part was particularly interesting, as sexual segregation has been studied mainly in vertebrates, especially ungulates^82^. In insects, however, this phenomenon has received only minor attention^83,84^. In general, differences in male and female behaviour may be attributed to three main hypotheses of sexual segregation: A) the reproductive-strategy hypothesis (intersexual differences in energetics and security), B) sexual dimorphism-body size hypothesis (body size dimorphism and different dietary requirements), or C) social-factors hypothesis (social mechanisms; intersexual aggression or territoriality)^85^. Compared to males, females typically invest a larger amount of resources in reproduction^86^. Therefore, according to the first two hypotheses, females should be more selective, as a higher energetic investment in reproduction and the choice of high-quality habitats for their offspring should force them to track resources more strictly than males. In insects, however, a predominant factor that affects female distribution patterns is often male harassment^87,88^, indicating that the social-factors hypothesis may play a dominant role. Indeed, in both species of diving beetles, there is only minor sexual size dimorphism but strong sexual antagonism. This is evidenced by the development of sophisticated grasping devices in males, in the form of circular, sucker-shaped adhesive setae on their tarsomers, which is counteracted by multiple modifications to the female dorsal cuticle^89^. Female morphological “anti-grasping” devices are quite rare in animals^54^, highlighting the importance of male harassment in habitat selection of diving beetles. Male harassment has multiple negative effects on female fitness, including direct mortality during prolonged mating events^89^, increased energy costs^89,90^, reduced foraging effort^91^, female fecundity^92^, and access to higher-quality food^93^. Consequently, males often occupy higher-quality habitats with abundant food sources, while females use lower-quality habitats to avoid harassment^88,94^. The lack of differences between male and female behaviour in our study was therefore unexpected.

In backswimmers, both sexes were consistent in their selection (i.e., there was no sexual segregation). This pattern was not surprising as they exhibit only minor sexual dimorphism^56,57^. Therefore, the role of male harassment is likely of lower importance, as it is probably obviated by the conspecific/heterospecific attraction discussed above. This may be corroborated by the fact that in backswimmers, dispersal tendency is independent of sex ratio^57^.Importantly, in our experiment, all pools were relatively small and similar in area. Therefore, we could not capture preferences based on area and overall habitat dimensions. Furthermore, some species may react inappropriately or atypically in small habitats, or when habitat availability is limited compared to natural conditions^95^. Nevertheless, previous studies have shown that the study taxa may not disperse great distances if multiple aquatic habitats are readily available nearby^96,97^. On the other hand, the ponds that are in close proximity may be functionally disconnected when the fish are present^98^.

Our study extends the current knowledge of habitat selection of aquatic insects in response to a top predator by examining differences in individual behaviour in relation to habitat specialisation and sex. We found different strategies for habitat selection in backswimmers and diving beetles, but there were no differences between specialists and generalists or between the sexes. At the population level, backswimmers showed clear habitat preferences; individual habitat selection decisions were consistent and possibly driven by conspecific/heterospecific attraction. In contrast, the selection of diving beetles was primarily density-dependent, regardless of fish presence or vegetation structure. Unlike backswimmers, individual beetle decisions were generally inconsistent, suggesting the opportunistic nature of their behaviour. The results of this study reinforce the importance of individual habitat selectivity for the colonisation of aquatic ecosystems, while suggesting a range of mechanisms driving animal behaviour. Nevertheless, individual variability in habitat selection remains largely unexplored in insects, and much of our discussion has been speculative at this point. Future experiments examining the effect of the presence of conspecifics/heterospecifics and resource availability on individual variability in habitat selection of other taxa, as well as focusing on individual differences in habitat selection by specialists and generalists under the consumptive effect of fish, may shed light on how widespread the patterns found in our study are, and gain a better understanding of how individuals combine information from multiple sources when selecting habitat.

## Supplementary Information

Supplementary Information.

## Data Availability

The data that support the findings of this study are permanently archived in the figshare data repository under the link https://doi.org/10.6084/m9.figshare.20440056.
