# Supplementary material for: Individual variability in habitat selection by aquatic insects is driven by taxonomy rather than specialisation
Source: Sci Rep. 2022 Dec 1;12:20735. doi: 10.1038/s41598-022-25363-3 (PMC9715563; doi:10.1038/s41598-022-25363-3)

**Supplementary Information** for

# Individual variability in habitat selection by aquatic insects is driven by taxonomy rather than specialisation

Authors: Hana Šigutová, Martin Šigut, Aleš Dolný, Filip Harabiš

**Correspondence:** Hana Šigutová: hana.sigutova@osu.cz; Filip Harabiš: harabis@fzp.czu.cz

**This file includes:**

Figures S1–S4

**Figure S1.** Treatment with submersed and floating macrophytes (A), treatment with submersed, floating + littoral macrophytes (B), marked individuals of *Notonecta glauca* and *N. obliqua* (C), marked individuals of *Acilius sulcatus* (D), overall view on one of the blocks €.

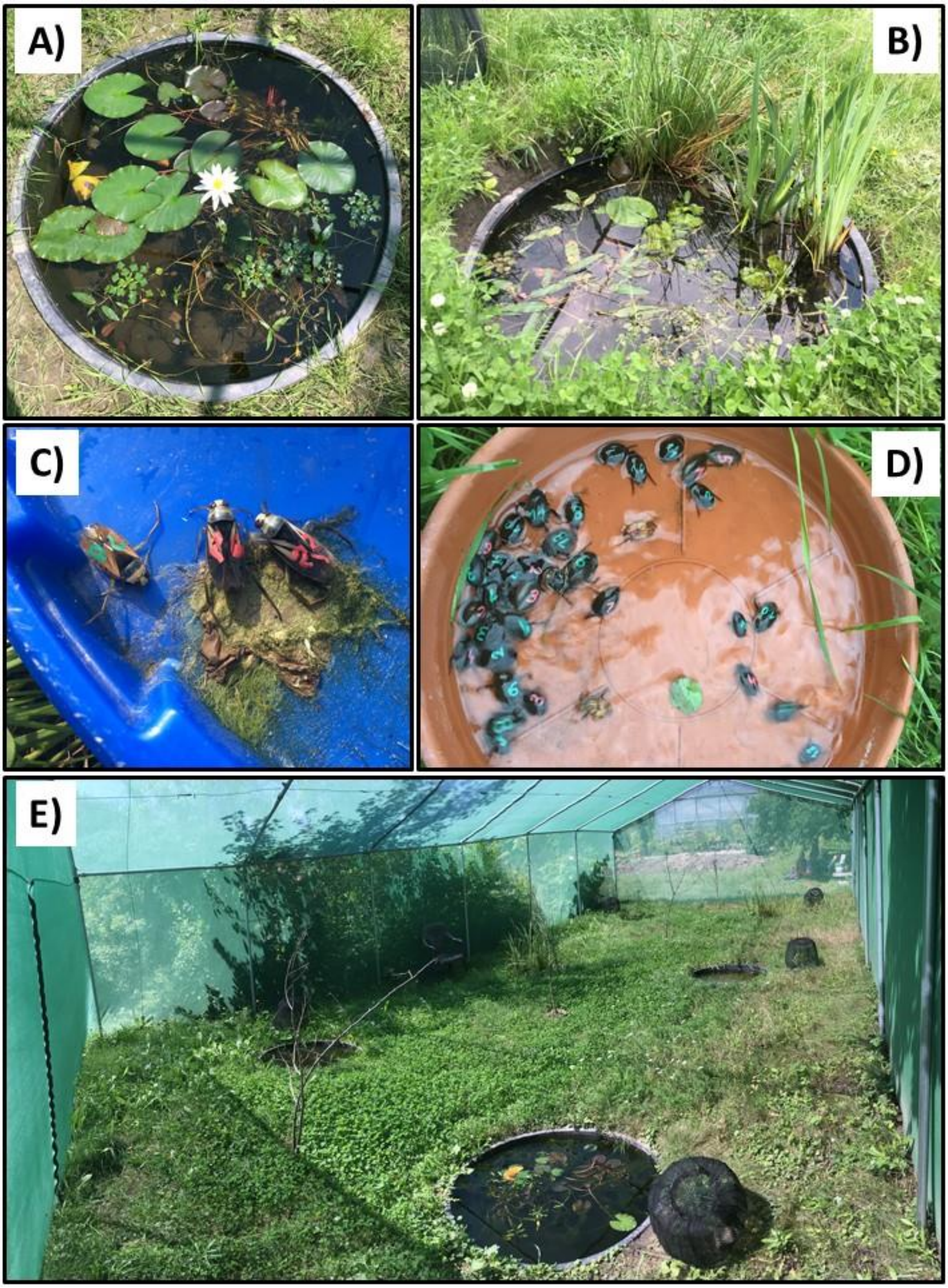

**Figure S2.** Habitat change frequency between males and females. Overall proportion of individuals that changed pool type between individual sampling events in backswimmers (*Notonecta glauca*, *N. obliqua*), and diving beetles (*Dytiscus marginalis*, *Acilius sulcatus*). Differences between males and females were insignificant in all study species.

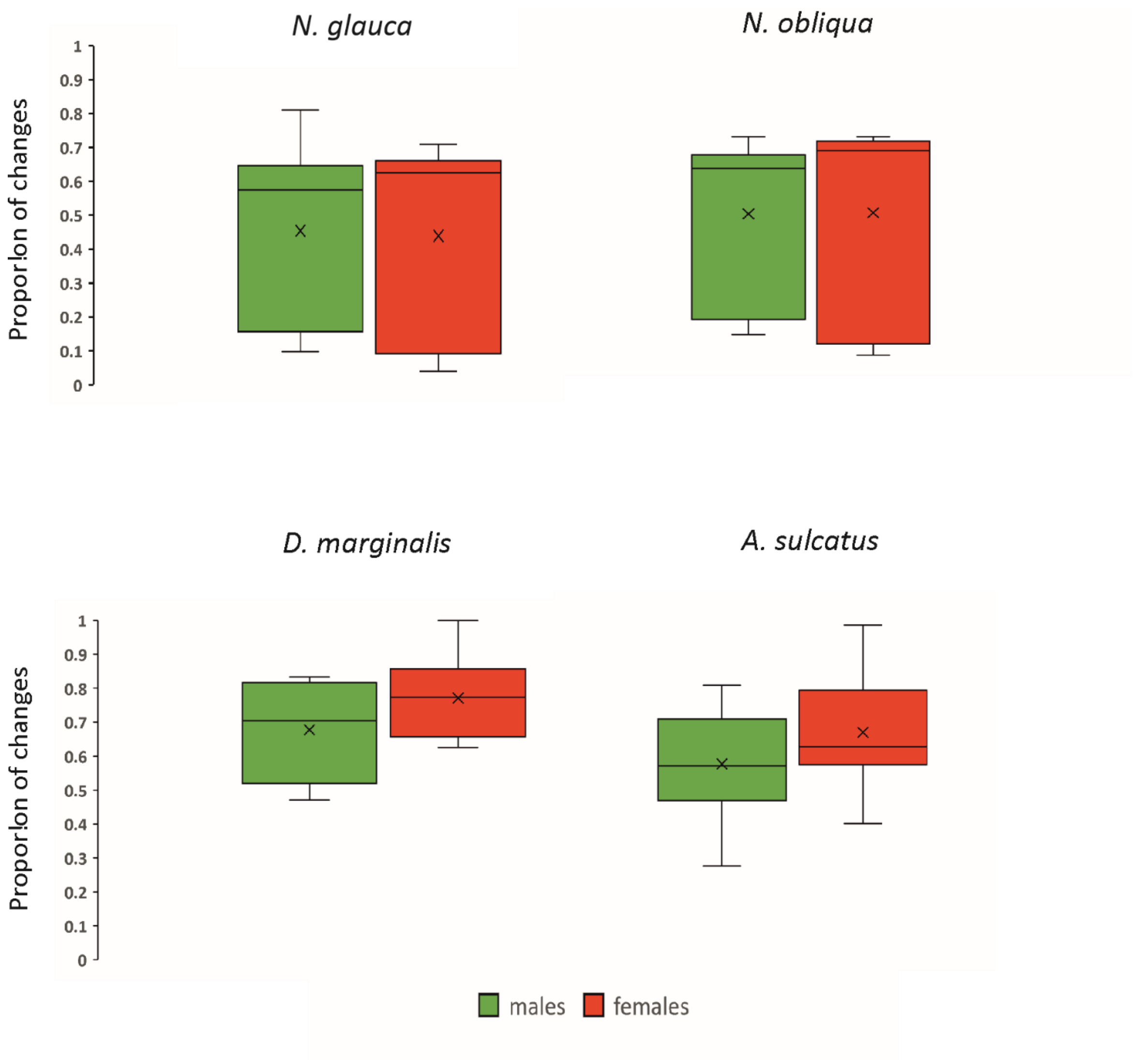

**Figure S3. Number of habitat types individuals selected over the whole experiment.** Individuals could select among six habitat types (fish or fishless pools with different vegetation structure). *Notonecta glauca* and *Dytiscus marginalis* represent habitat generalists, while *N. obliqua* and *Acilius sulcatus* are species specialized in fishless habitats, vulnerable to fish predation. Illustrations were created by the authors.

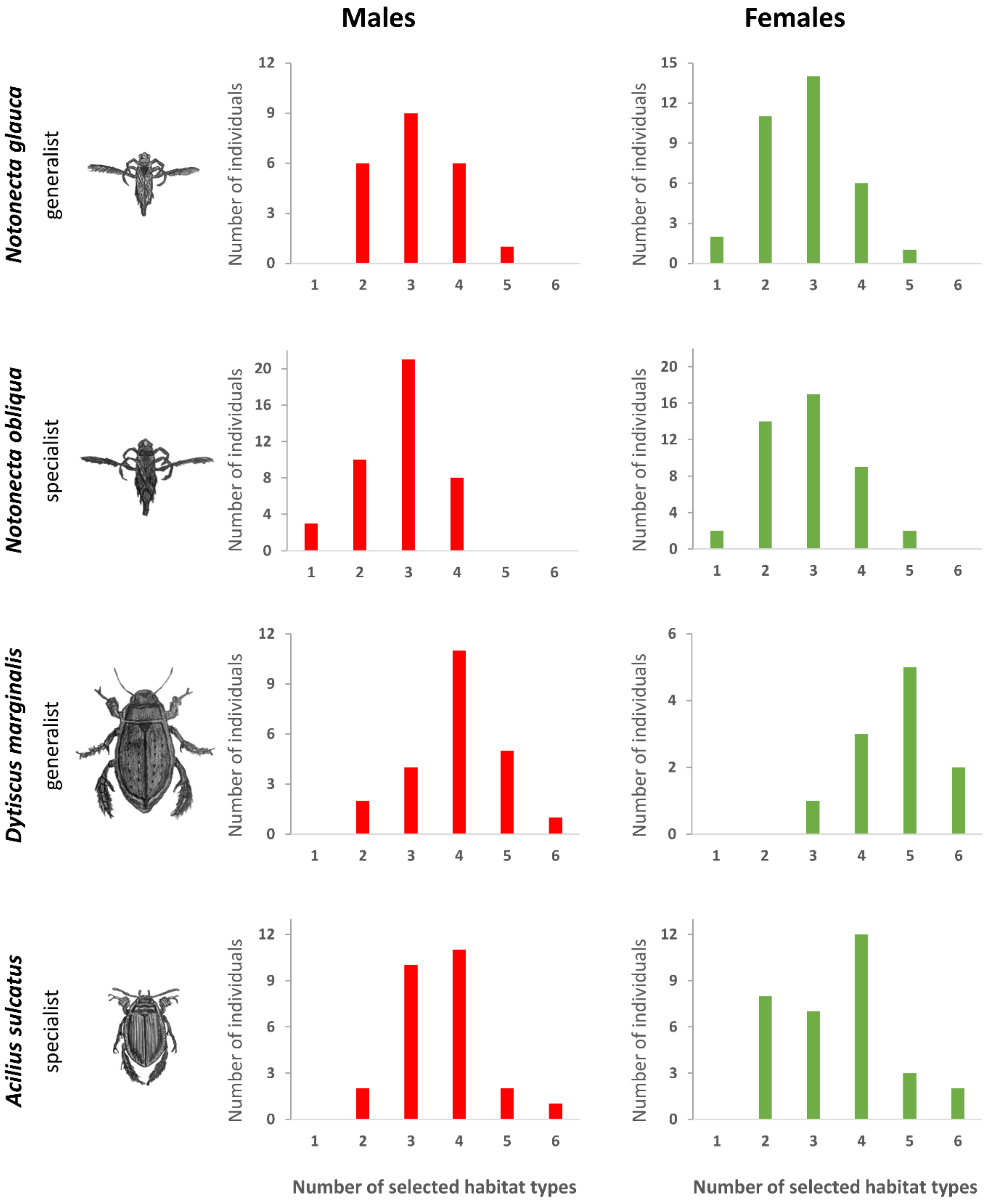

**Figure S4.** Habitat selection history of backswimmers (*Notonecta glauca*, *N. obliqua)*, and diving beetles (*Dytiscus marginalis*, *Acilius sulcatus*) in response to fish and different vegetation structure (no macrophytes; submersed and floating macrophytes; submersed, floating + littoral macrophytes). In each table, the numbers in rows represent sampling occasions, while numbers in columns represent individuals of respective species. Data show history of all individuals that were captured in at least half of all the sampling events, regardless of block. Illustrations were created by the authors.

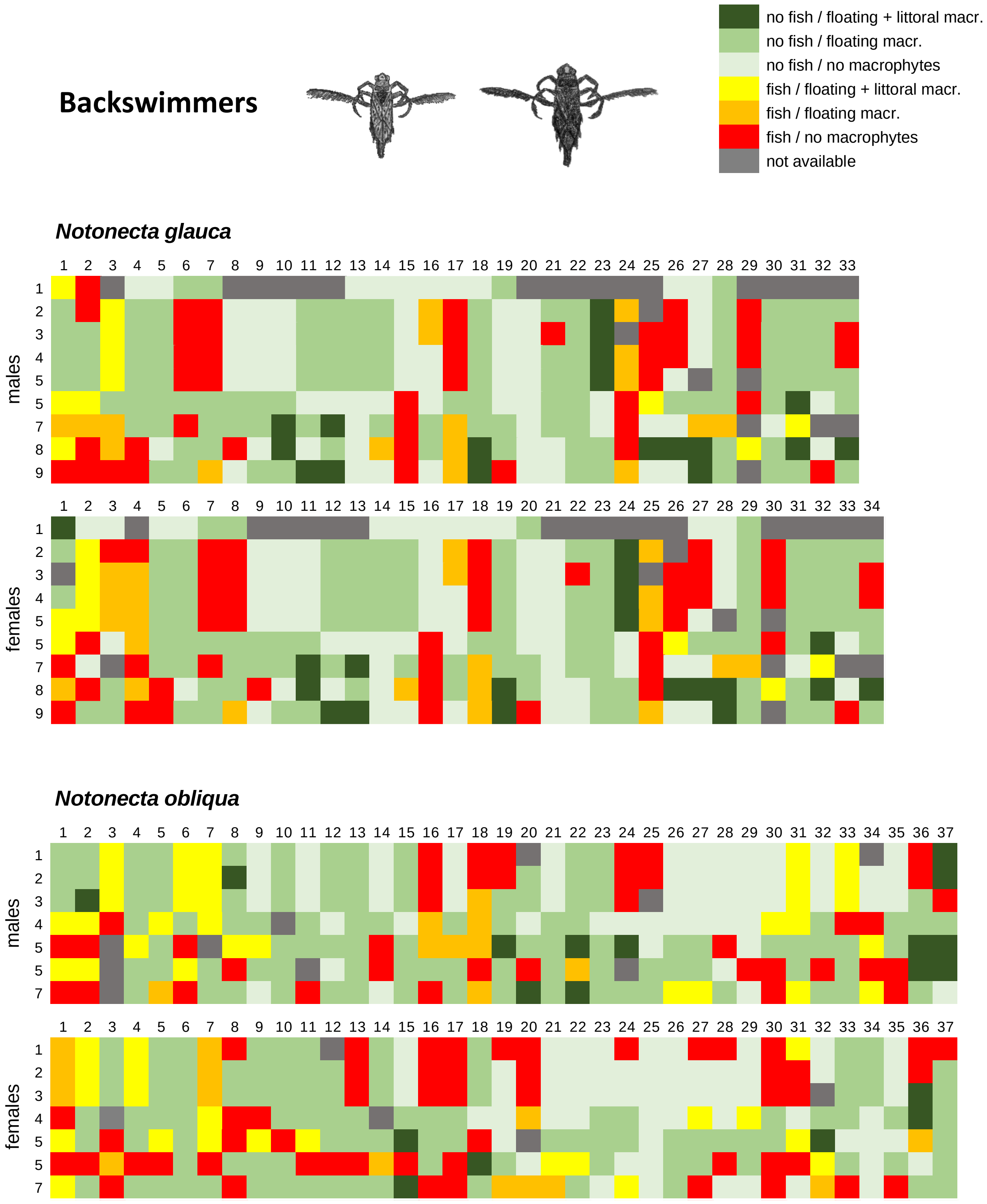

# Diving beetles

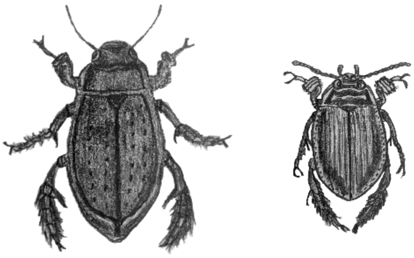

- no fish / floating + littoral macr.
- no fish / floating macr.
- no fish / no macrophytes
- fish / floating + littoral macr.
- fish / floating macr.
- fish / no macrophytes
- not available

## *Dytiscus marginalis*

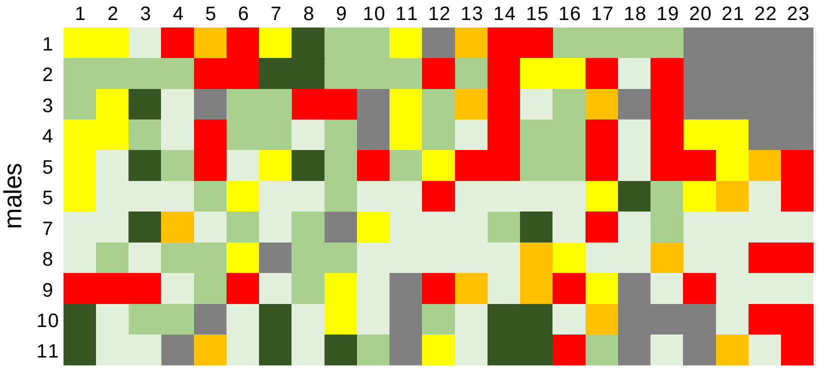


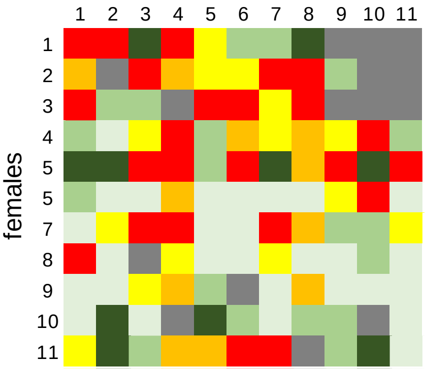

***Acilius sulcatus***

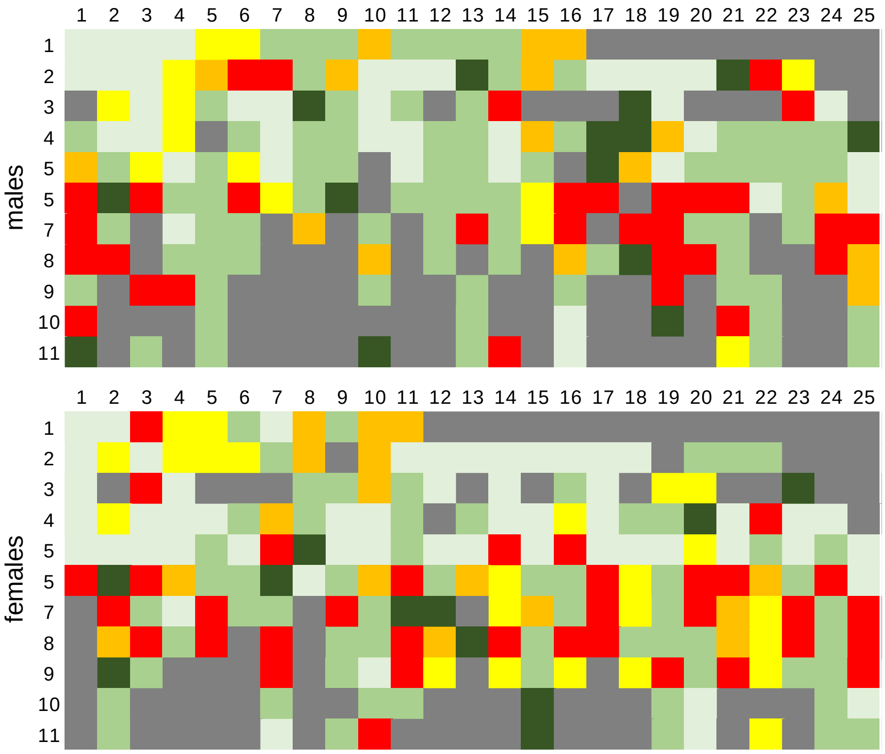

Supplement: Supplementary file 1 — Supplementary Information. [file 41598_2022_25363_MOESM1_ESM.pdf]
